# Pathology podcasts: a growing educational tool

**DOI:** 10.1016/j.acpath.2025.100227

**Published:** 2025-11-03

**Authors:** Sarah Mohamed, Casey P. Schukow, Jennifer Worthy, Dennis Strenk, Nicole R. Jackson, Sanam Loghavi, Xiaoyin Sara Jiang, Andrew M. Bellizzi, Michael A. Arnold

**Affiliations:** a Department of Undergraduate Medical Education, Kiran Patel College of Osteopathic Medicine, Nova Southeastern University, Fort Lauderdale, FL, USA; b Department of Pathology, Corewell Health William Beaumont University Hospital, Royal Oak, MI, USA; c Lexington Family Medicine, University of South Carolina, West Columbia, SC, USA; d Wisconsin Diagnostic Laboratories, Milwaukee, WI, USA; e Department of Laboratory Medicine and Pathology, University of Washington, Seattle, WA, USA; f Department of Hematopathology, Division of Pathology/Laboratory Medicine, The University of Texas MD Anderson Cancer Center, Houston, TX, USA; g Department of Pathology, University of South Florida and Ruffolo, Hooper, & Associates, Tampa, FL, USA; h Department of Pathology, University of Iowa Hospitals and Clinics, University of Iowa Carver College of Medicine, Iowa City, IA, USA; i Department of Pathology, University of Colorado and Department of Pathology and Laboratory Medicine, Children's Hospital Colorado, Anschutz Medical Campus, Aurora, CO, USA

**Keywords:** Curriculum, Medical education, Pathology, Pathology education, Podcasting, Presentation, Social media

## Abstract

Podcasts serve as an easy, affordable, and on-the-go experience for listeners of all types, particularly those casually broaching a new subject they are interested in learning more about. Podcasting as a tool for pathology education has had a growing reach for several years, and has gained traction as an academic work product for medical education, promoting journals and articles, and attracting trainees to pathology. Yet, there is sparse academic literature reviewing the topic. In this article, we aim to provide a qualitative synthesis of peer-reviewed and other online sources, with objectives including introducing podcasting, reviewing its role in medical education, and exploring issues unique to pathology. National podcasting trends, advantages, and limitations of podcasts in pathology will be covered. Additionally, a comprehensive list of pathology podcasts, quantitative analysis of select podcast downloads, and practical steps for pathologists interested in creating their own podcasts will also be provided.

## Introduction

Social media (SoMe) platforms like Twitter/X and Meta (e.g., Facebook and Instagram) allow users to connect, collaborate, network, and disseminate information globally. SoMe has been referred to as a “double-edged sword” in medical education (MedEd), as the positives of SoMe might be accompanied by medicolegal and ethical criticisms such as superficial learning, professionalism, ownership protection of content, privacy concerns, and misinformation distribution.^1^ Despite these and other criticisms,^2^ SoMe and other web-based platforms have augmented the reach of MedEd innovation and the delivery of fact-based information to trainees, medical students, and the public, especially during the coronavirus-2019 (COVID-19) pandemic and socially distanced learning.^3^

In recent years, pathology has become just one field in which SoMe has become a powerful tool for MedEd.^4^ The number of peer-reviewed articles regarding the application of SoMe to pathology MedEd continues to rise; a recent systematic review by Flippo et al.^5^ included 54 published articles (as of December 2022) for analysis. However, one modality with sparse medical literature addressing its utility in pathology is podcasts, which are often shared across other SoMe platforms. This review aims to provide a brief primer on podcasting in pathology via a qualitative analysis and synthesis of peer-reviewed and non-peer-reviewed online sources, including (1) what podcasting is, (2) the advantages and limitations of podcasting in pathology, with ideas for future research, (3) what pathology podcasts currently exist, and (4) how pathologists can start their own podcasts.

## What is podcasting?

Podcasts are digital audio or video files that can be downloaded or streamed from a website, mobile app (i.e., on a smartphone), or other platform. Some people refer to podcasts as SoMe, while others may call them “new media” since podcasts feature a one-way flow of communication like radio programming, rather than a two-way flow.^6^ Regardless, podcasts typically consist of a host and a guest discussing a chosen topic or set of related ideas in a casual setting with varying degrees of formality. Each audio or video file is considered a single episode, and lengthier topics may be covered in a multi-episode series, similar to a television series. The complete set of episodes is released together under one common title for the podcast show. Each episode is sometimes divided into different segments of interrelated themes under the more general topic of the episode, usually titular. Formatting can vary by episode or by show. A seasoned show host will point out transitions between topics or segments during the episode as well as set the tone and layout for the episode at the beginning. In addition, the host will typically introduce themselves and any guests present, including any sponsorships by private entities, while providing catchy music or “jingles”. Advertisements are often shared during mainstream commercial podcast episodes. In 2024, advertisers were projected to spend approximately $2.28 billion on commercial podcasts, which was a 15.9 % increase from 2023.^7^ However, most pathology podcasts focused on academic topics avoid distracting advertisements and elect not to generate ad revenue.

Podcasts have become one of the most popular platforms for entertainment and communication. They reach a wide audience as audio recordings can be listened to anywhere in the world at any time convenient for the listener via an internet connection, or downloaded in advance when the listener anticipates traveling to an area where the internet is limited. Access to podcasts is almost universal, as long as the user has a device to listen to the podcast. Many podcasts are generally uploaded to a show's website and shared to other platforms using a Really Simple Syndication (RSS) feed that can be accessed by platforms like Spotify (https://open.spotify.com/), Apple Podcasts (https://podcasts.apple.com/), YouTube (https://www.youtube.com/), and Amazon Music (https://www.amazon.com/music/), without cost to the user, though advertisements may run between episode segments. All of these platforms include options for paid upgrades without ads or access to premium shows and episodes, but their podcast libraries have a free option for listeners.

In 2020, Rodman and Trivedi^8^ outlined the history of podcasts and their efficacy in MedEd, including the vision for a “global podcasting curriculum” that would involve “interactive, high-quality, and up-to-date” digital information available from any mobile or smart device, at any time, by 2025. i.e., an increased emphasis on virtual, flexible, learner-centered education, which is already being described in pathology undergraduate- and graduate-medical education (UME and GME, respectively).^9^, ^10^, ^11^ In 2022, Kelly et al.^12^ identified 44 studies on the topic via a scoping review of PubMed and Embase databases from June to July 2020. Educational outcomes were reported in 38 studies, with multiple other studies showing positive learner reactions and attitudes toward podcasts, including value for podcasts portability, efficiency, and combined educational and entertainment value. Though systems change or patient outcome differences were not reported in any of the identified podcasts, the authors identified 11 studies that showed improved documentation skills among medical students and enhanced reporting practices in both residents and physicians.

## Advantages of podcasting in pathology

For pathologists, podcasts are an easy and inexpensive modality to share their knowledge. As research and development in pathology expands, podcasts continue to grow as a platform to rapidly disseminate updates in the field, with many pathology podcast shows publishing monthly or even weekly episodes. This serves as a free supplement for getting the most up-to-date information. Many pathology podcasts are aimed at those interested in pursuing or who are new to the specialty, while others are intended for pathologists working in the field for many years. Subject matters range from hard science and hot topics to ethics and considering the future directions of the study of pathology and artificial intelligence. Some are geared towards reviewing board exam material, again offering another free option for students of pathology MedEd. The low barrier to entry, easy accessibility, and typically zero additional costs make pathology podcasts ideal. However, it should be noted that certain podcasts may only be available on podcasting platforms that require a monthly or yearly subscription for complete access.

Unlike live lectures, podcasts can be paused, slowed, sped up, or rewound by the listener for their own understanding. Many recorded lectures are otherwise used as podcasts: many learners listen to them while commuting or at home. However, unlike lectures, the relaxed, unscripted nature of many podcasts provides a low-stress learning environment for busy learners, allowing them to take charge of their education (i.e., self-directed learning, which is an important skill for medical students and trainees to develop for successful lifelong learning).^13^ Research in undergraduates and primary school children has shown that calming and comfortable environments, which traditional learning spaces like classrooms or lecture halls may not always be, can increase recall and facilitate learning.^14^

Overall, less-structured MedEd modalities such as podcasts may be helpful for enjoyable, lifelong learning by pathologists (at all career stages, including medical students, residents, and fellows), practitioners of fields that are adjacent to pathology, laboratory medicine-associated professionals (including pathology assistants and histotechnicians), and the general public. Many podcasts focus on hot topics of public interest to sustain such lifelong learning; the same is true in pathology. Though these topics may go above and beyond structured core essentials required for school or training (in the setting of UME and GME programs, respectively), it should be noted that podcasts may be better suited for everyday use in busy practice or learning settings as adjunctive tools to remain up-to-date with specific topics of interest (e.g., surgical pathology, molecular testing, blood banking, microbiology). Though continued research is needed to determine optimal learning environments for pathology podcasts in medical school, residency, fellowship, and practice settings, the general concept of comfort and empowerment toward enhanced and self-directed learning likely remains true. Additionally, the podcast's role in each environment mentioned above should be vetted, as current literature is sparse.

## Possible limitations with suggestions for future research

The discussion surrounding the utilization of podcasts in pathology MedEd has yet to be adequately explored or quantified. Data such as audience growth trends, trends in listening and publication of pathology subtopics, and how and when listeners are multitasking while listening to podcasts should be made publicly available and collated to shed more light on how these podcasts are increasingly becoming an important tool in MedEd. Though proof-of-concept has been established as aforementioned, further research should focus on the efficacy of pathology podcasts as an adjunct resource for MedEd in comparison to other forms of SoMe and traditional didactic-style teaching, including knowledge retrieval and application over time.

Despite the advantages of podcasts in MedEd, there are also some field-specific concerns among pathologists. Pathology is a highly visual discipline, making listening to an audio podcast less desirable when compared to audiovisual content such as PathElective.com (https://www.pathelective.com/),^15^, ^16^, ^17^ pathCast (https://www.youtube.com/c/pathCast),^18^^,^^19^ and online content from Dr. Jerad Gardner (https://kikoxp.com/posts/5084/public).^20^ Thus, a pathologist may be skeptical if it is even possible to sufficiently learn and understand a pathology concept via an audio podcast. Furthermore, variation in terminology used to describe tumors and tissues can present challenges, as there can be multiple names for the same entity.^21^ Moreover, some pathologists have developed their own verbal descriptions to articulate the pathology they are illustrating. This has the potential to make the seamless sharing of information through podcasting unclear.

Pathology is a highly visual field. While an audio-only podcast format at first might appear to present a limitation for presenting pathology material, pathologists are adept at translating visual information into words to generate reports in their clinical practice. It should be noted that certain pathology topics focus more on conceptual domains rather than visual ones, such as clinical pathology, forensics, digital, and informatics. As mentioned previously, topics such as these continue to be relevant in modern practice and may be worthwhile for the general public to be exposed to in support of advocating in-practice pathologists and other laboratory medicine professionals. In addition, while podcasts were originally developed as audio-only tools, the term has evolved, and it is increasingly common for podcasts to be multimodal and include a video component.

## What pathology podcasts are already out there?

Although in its relative infancy, there are over 30 pathology-centered podcasts available for download listed in Table 1, with clickable hyperlinks provided. In the following paragraphs, specific pathology podcasts will be highlighted as specific examples, though we strongly encourage readers to explore these and the other podcasts listed in Table 1.

**Table 1:** Alphabetized list of pathology podcasts.^a^^,^^b^

Title	Host(s)^c^	Website Link	Topic(s)
*Archives of Pathology & Laboratory Medicine AI-Generated Podcasts*	CAPTodayOnline	https://www.captodayonline.com/archives-of-pathology-laboratory-medicine-ai-generated-podcasts/	CAP journal updates; select articles discussed using AI generated voices
*Audio Insights*	Mayo clinical laboratories	https://podcasts.apple.com/us/podcast/audio-insights/id1245234717	Featuring departments and faculty of Mayo clinical laboratories
*BloodBankGuy Essentials Podcast*	Chaffin	https://www.bbguy.org/podcast/	Blood bank and transfusion medicine
*Beyond the Scope*	DPA	https://digitalpathologyassociation.org/dpa-podcast-beyond-the-scope	Hot topics in digital pathology, including research, technology, and workflows
*Blood Podcast*	ASH	https://ashpublications.org/blood/pages/blood_podcast	Hematology and hematopathology
*CAPcast*	CAP	https://soundcloud.com/pathologists	Pathology advocacy, career building, professionalism, diversity, equity, and inclusion
*Clinical Chemistry Podcast*	ADLM	https://myadlm.org/science-and-research/clinical-chemistry/clinical-chemistry-podcasts	Clinical chemistry
*Deadman Do Tell Tales*	Croom, Taylor	https://www.deadmendotellpodcast.com/	De-mystifying forensic pathology
*Deeper Levels*	Banet	https://podcasts.apple.com/us/podcast/deeper-levels/id1505437181	Choosing pathology as a career, medicine, science
*detroit'sdailydocket*	WCMEO	https://detroitsdailydocket.buzzsprout.com/	Forensic pathology
*Dialogues on AI Digital Pathology*	HistoIndex	https://anchor.fm/histoindex/episodes/Official-Trailer-Special-Episode-PART-4---Conversation-with-KOL-Hepatologists-and-Pharma-Thought-Leaders-in-NASH-e122ril	Artificial intelligence and digital pathology in finding treatments for non-alcoholic steatohepatitis
*Digital Pathology Today*	Anderson	https://www.digitalpathologytoday.com/	Discussions with industry leaders and academic topics on digital pathology
*Digital Transformation in Pathology*	Zuraw	https://visiopharm.com/resources/?type[]=podcast	Artificial intelligence and digital pathology
*Diversify in Path*	Williams	https://diversifyinpath.buzzsprout.com/	Embracing diversity in pathology
*Follow your Path!*	Abid, Herman, Schukow, Fernandez, Tariq	https://followyourpath412.podbean.com/	Interviews with pathologists who represent different fields in and pathways to pathology
*Inside the Lab*	ASCP	https://www.ascp.org/content/learning/inside-the-lab#	Current topics in pathology and laboratory medicine
*Knife After Death*	Wolfe	https://knifeafterdeath.podbean.com/	Death, decay, and forensic pathology
*Lab Calling Podcast*	Lab Calling	https://podcasts.apple.com/mv/podcast/lab-calling-podcast/id1509492008	Clinical laboratory medicine
*Labmind*	Jackson	https://podcasts.apple.com/us/podcast/labmind/id1410853605	Diagnostic laboratory medicine
*Laborastories*	ADLM	https://myadlm.org/community/podcast	
*Meet the Microbiologist*	Hagen, ASM	https://asm.org/Podcasts/MTM	Microbiology
*Micro Waves Podcast*	RCPA	https://www.rcpa.edu.au/Library/Publications/Micro-Waves-Podcast	Education and awareness in pathology
*ModPath Chat*	USCAP	https://www.nature.com/modpathol/modpath-chat	Latest science, technology, and development in pathology
*Pathology Made Non-Toxic*	Rakeesh	https://www.spreaker.com/show/pathology-made-non-toxic-by-dr-r	Basics of pathology
*People of Pathology Podcast*	Strenk	https://peopleofpathology.podbean.com/	Interviews with pathologists about what they do in their fields
*The Pediatric and Developmental Pathology Podcast*	*Pediatric and Developmental Pathology*	https://pedidevpath.podbean.com/	Pediatric pathology
*The Digital Pathology Podcast*	Zuraw	https://digitalpathologyplace.com/podcast/welcome-to-the-digital-pathology-podcast/	Exploring medical and scientific advancements in digital pathology
*The Grenz Zone*	Kolb	https://grenzzonederm.com/	Dermatology and dermatopathology
*The Hematologist Podcast*	ASH	https://ashpublications.org/thehematologist/pages/podcasts	Hematology and hematopathology
*The Journal of Pathology Podcasts*	The journal of pathology	https://onlinelibrary.wiley.com/page/journal/10969896/homepage/podcasts_from_the_journal_of_pathology.htm	Pathology journal content
*The Pathologists Cut Podcast*	RCPA	https://www.rcpa.edu.au/Library/Publications/The-Pathologists-Cut-Podcast	Critical work of pathologists and its integration in medicine and healthcare
*The Pathology Grand Tour*	The pathologist	https://thepathologist.com/podcasts/the-pathology-grand-tour	Highlights about different pathology fields
*The PathPod Podcast*	Jiang, Mirza, C. Arnold, M. Arnold	https://pathpod.podbean.com/	Roundtable pathology interviews, quizzes, tips, and more!
*The SCVP's Podcast*	SCVP	https://scvp.podbean.com/	Cardiovascular pathology
*This Pathological Life*	Davis, Brown	https://www.clinpath.com.au/clinicians/education/podcast-this-pathological-life/	Stories and discussion about different pathology conditions
*Throwback Thursday with Dr. Fred Silva*	Arkana laboratories	https://podcasts.apple.com/us/podcast/throwback-thursday-with-dr-fred-silva/id1242963310	Renal pathology

Abbreviations: ADLM, Association for Diagnostics and Laboratory Medicine; AI, Artificial Intelligence; ASCP, American Society for Clinical Pathology; ASH, American Society of Hematology; ASM, American Society for Microbiology; CAP, College of American Pathologists; DPA, Digital Pathology Association; RCPA, Royal College of Pathologists of Australasia; SCVP, Society for Cardiovascular Pathology; USCAP, United States and Canadian Academy of Pathology; WCMEO, Wayne County Medical Examiner's Office.

a As of June 2025.

b This list is composed via a qualitative review of pathology podcasts online and via mobile streaming applications using the search term “pathology”. Certain podcasts were added based on “word-of-mouth” recommendations, too. Not all podcasts may still be active, and hosts may have changed. Additionally, as there are so many podcasts out there, some podcasts may be excluded (not intentionally, of course). Further peer-reviewed studies should be pursued for more comprehensive quantification as necessary. Follow-up studies should explore the continued growth and identification of pathology (and laboratory medicine) related podcasts, as this is a relatively nascent topic in academic medicine.

c Listed by either individual hosts (last name) or abbreviations of companies/organizations (see below).

Given their easy accessibility to members and non-members, national pathology organizations have taken to podcasting. The College of American Pathologists (CAP), a 501(c)(6) nonprofit physician-membership organization of approximately 18,000 board-certified pathologists, fosters and advocates for best practices in pathology and laboratory medicine. CAP began its podcast in September 2014, the CAPcast (https://soundcloud.com/pathologists). One of the earliest podcasts in pathology and laboratory medicine, CAPcast features interviews with leading pathologists on current issues impacting pathology and laboratory medicine. Having aired over 370 episodes to date, CAPcast also features series by topic, such as “Biorepository” and “New-in-Practice,” which allow listeners to learn from multiple episodes centered on the same topics. While tailored to its membership, CAPcast is open and freely accessible to all, and, similar to other podcasts, promotes episodes on SoMe in addition to email directly to its membership. Other professional organizations, such as the American Society for Clinical Pathology (ASCP) and the United States and Canadian Academy of Pathology (USCAP) have similarly produced their own podcasts, “Inside the Lab” and “Modern Pathology”, respectively.

## Quantitative analysis of representative pathology podcasts

The traditional format of podcasting consists of a host conducting serial interviews with different guests, as exemplified by the People of Pathology podcast (https://peopleofpathology.podbean.com/). Hosted by creator Dennis Strenk, a pathologist's assistant, this podcast has weekly episodes where listeners learn more about the person behind the laboratory worker. With over 200 episodes and 104,000 downloads since its first airing in December 2019, People of Pathology has touched on topics including education, teaching, recruitment, mindfulness, gross specimen handling, emerging technology, rural and international medicine, SoMe, and more. Guests include notable or up-and-coming pathologists, pathologist assistants, medical laboratory specialists, surgeons, forensic anthropologists, and autopsy technicians. In doing so, the podcast has essentially generated a list of potential mentors and resources for those planning a career in a specialty directly or indirectly related to all fields of pathology and laboratory medicine.

Created by pathologists Drs. Sara Jiang, Michael Arnold, Christina Arnold, and Kamran Mirza, PathPod (https://pathpod.podbean.com/) is a trainee-centered pathology podcast that has produced more than 88 episodes with over 95,000 downloads since its first airing in April 2020. PathPod deviates from traditional podcasts by using a variety of hosts to represent a range of voices in the field. PathPod has also used multiple episode types in addition to occasional special episodes. “Beyond the Scope” episodes follow the more traditional format of a host or hosts interviewing a guest or guests on their practice and interests outside of pathology, while “Around the Scope” episodes gather a small group of pathologists and/or pathology trainees with similar areas of interest to discuss their practice, experiences, and/or a shared publication. The application of immunohistochemistry (IHC), a challenging practice for trainees to master, is discussed in “IHC Talk” episodes centered on hematopathology, bone and soft tissue pathology, SARS-CoV-2, managing IHC laboratories, and proposed legislation that impacts the practice of pathology. Balancing academia and admixed with the aforementioned categories are “News Edition” episodes that give special attention and discussion to important and timely topics, including the COVID-19 pandemic, diversity, wellness, and inclusive curriculum development, and “Quiz Show” episodes that lead with a fun, laid-back approach to getting to know guests.

**Fig. 1:**
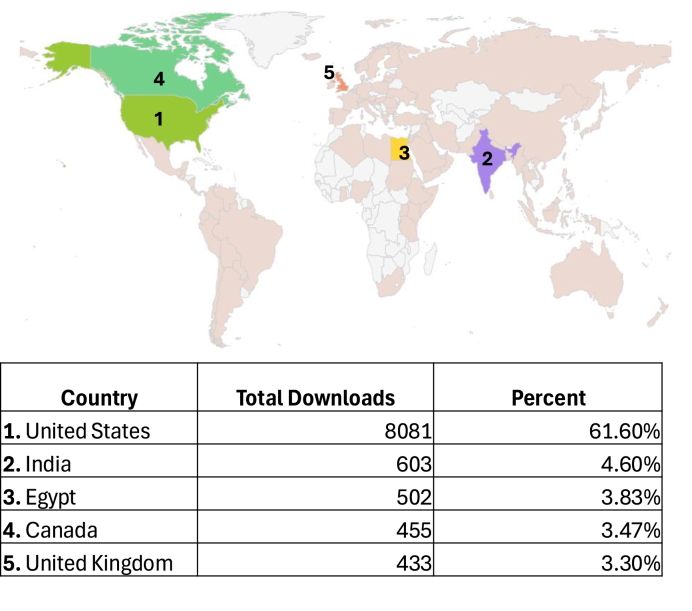
Where listeners downloaded PathPod podcast episodes from over 24 months (ending January 2025).

Table 2 and Fig. 1 show how most listeners have downloaded PathPod episodes and available location information over 24 months ending January 2025, including data for 13,119 downloads. Of note, location information may not be available for some users based on device settings and not included in the data displayed by hosting services. The most common platform listeners have downloaded PathPod episodes are from Apple Podcasts (n = 7808/13119, or 59.52 %) and Spotify (n = 1171/13119, or 8.93 %), followed by Google Chrome (n = 1087/13119, or 8.29 %), Safari (n = 323/13119, or 2.46 %), and others. This supports the importance of having one's podcast on multiple popular podcasting apps to increase access to the podcast. Additionally, even though the top country with the most downloads during this period was the United States (n = 8081/13119, or 61.60 %), podcast episodes were downloaded in 70 other countries throughout the world (total n = 5038/13119, or 38.40 %), including India, Egypt, Canada, and the United Kingdom. A complete table showing the remaining countries with total downloads and relative percentages is in Supplemental Table 1. This data suggests that this specific podcast, like most other podcasts, achieved significant global reach and supports the capability for podcasts to provide global education to others, such as in developing countries. Generally, downloaded data may have limited utility, namely that one user can download an episode multiple times, and that just because an episode is downloaded does not necessarily mean it was listened to. Overall, these data suggest that there can be a potentially positive global impact with podcasts in pathology and that future in-depth studies should be pursued to gauge this outcome, not just by this pathology podcast, but by other pathology podcasts as well.

**Table 2:** How listeners download PathPod podcast episodes over 24 months (ending January 2025).

Client	Downloads (Total n = 13119)	Percentage of Total
Apple podcasts	7808	59.52 %
Spotify	1171	8.93 %
Google Chrome	1087	8.92 %
Safari	323	2.46 %
Overcast	312	2.38 %
PodbeanApp	275	2.10 %
Edge	233	1.78 %
Google podcasts	192	1.46 %
Lavf	188	1.43 %
Firefox	109	0.83 %
CFNetwork	103	0.79 %
iTunes	91	0.69 %
Pocket Casts	84	0.64 %
PodcastAddict	82	0.63 %
CastBox	74	0.56 %
Downcast	17	0.13 %
Mobile Safari UIWebView	17	0.13 %
AntennaPod	15	0.11 %
Snipd	15	0.11 %
Stitcher	12	0.09 %
Android Player	11	0.08 %
DoggCatcher	9	0.07 %
Player FM	9	0.07 %
TuneIn	6	0.05 %
小宇宙 (“little Universe”)	5	0.04 %
Amazon music podcasts	3	0.02 %
Castro	3	0.02 %
Chromecast device	3	0.02 %
Deezer	3	0.02 %
Samsung free	3	0.02 %
aria2	2	0.02 %
Chrome	2	0.02 %
Facebook	2	0.02 %
iHeartRadio	2	0.02 %
Opera	2	0.02 %
Podimo	2	0.02 %
Twitter app	2	0.02 %
Alexa-enabled device	1	0.01 %
Microsoft edge	1	0.01 %
The Podcast app	1	0.01 %
Unidentified	839	6.40 %

## How to get started

**Fig. 2:**
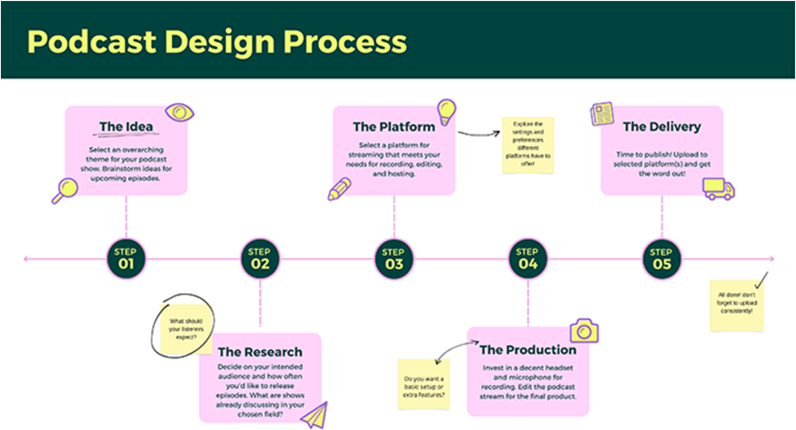
A hypothetical timeline of how to set up a pathology podcast (created using Canva).

While starting a podcast may seem daunting, it can be quite simple. An example hypothetical creation timeline for setting up a pathology podcast is shown in Fig. 2. The steps and set of products compiled below are based on the authors' own anecdotal experiences. A comprehensive review of podcast-related products is outside the scope of this article, although a future publication of this sort would be beneficial to academic literature. Additionally, obtaining podcast-related data for individual podcasting platforms, such as the number of subscribers and downloads for podcast episodes, is often straightforward since this functionality is often built into each platform. However, the level of difficulty for obtaining such data can vary depending on the platform, and a detailed overview of this is outside the scope of this article.

Due to the popularity of podcasts, many affordable services are available that make the process much easier. A few “all-in-one” platforms are available that allow you to do everything in one place, though one of the most popular is Spotify. This incorporates the recording software Riverside (https://riverside.fm/). Podcasters (people who create podcasts) can record, edit, and host all in one place. Similarly, SquadCast (https://squadcast.fm/) and Descript (https://www.descript.com/) can be used together for recording and editing. However, this still requires a separate hosting platform. Podbean (https://www.podbean.com/) offers recording, editing, and hosting together, and even has the capability for live streaming.

### Equipment

All podcasters need to get started is a microphone. While quality audio can be obtained with high-end microphones with lots of extra features (pop filters, etc.), one should not feel obligated to spend a ton of money to start a podcast. Even a reasonably priced headset with a microphone from a big box retailer can produce podcast-quality audio. It is not necessary to spend a lot of money on a microphone. Some high-end microphones are not suited for podcasting because they are too sensitive and pick up the slightest background noise. Even without high-end equipment, several audio editing platforms, such as Descript, offer audio enhancement tools that can automatically remove imperfections from recordings during editing.

### Recording platform

As mentioned previously, some recording platforms are now incorporated into hosting services, making it simple to use an all-in-one service. But if one prefers to record separately, Zoom (https://www.zoom.com/) is familiar to most hosts and guests. Several of the authors use Zoom for audio recording and find that the videoconference element facilitates a natural conversation, even if sometimes our guests refer to visual cues that are lost in the transition to audio-only. Zoom allows users to record audio on separate channels for each speaker, simplifying the editing process by preventing overlapping sound of different speakers that would occur in a single track recording. Remember to set the recording preferences in Zoom before starting a recording.

### Editing

Editing can be the most time-consuming element of creating a podcast, particularly if it's a podcaster's first time doing audio editing. Options range from waveform audio editors that are designed to be used with music or voice to editors specifically for podcasts that use transcribed text to help guide the editing process. Some of the authors of this article use Descript, a service that transcribes audio automatically and facilitates the editing of interviews, as it is similar to editing a Word processor document. Descript allows for video editing and transcription as well. It is now also possible to use Descript for editing Zoom recordings.

### Podcast Hosting

A podcast hosting server stores podcast files and creates a Really Simple Syndication (RSS) feed that facilitates distribution to the various podcast apps. There are several hosting options, which are generally quite affordable. A few of the most popular are Spotify for Creators (https://creators.spotify.com/), Libsyn (https://libsyn.com/), Buzzsprout (https://www.buzzsprout.com/), and PodBean (https://www.podbean.com/), each offering similar services. Each episode of a podcast will need an audio file, a text description, and cover art. Many services can assist with cover art, but podcasters can also use Canva (https://www.canva.com/) or PowerPoint do it themselves for free. There are size requirements for the image provided by each hosting site. Podcasters can always change the image later or use the same art for each episode, so don't let this step hold you up.

While podcasting may contribute positively to MedEd and public awareness of pathology, it can also raise the profile of podcast hosts and guests. An interview podcast is a powerful networking tool, as suggested in prior literature.^22^ It allows the host to build connections with the guests, which could be valuable later. Conducting interviews is great for practicing communication skills, too. Learning to craft a conversation that flows well is sometimes an art. This skill is transferable to pathology. It is also helpful in developing the speaking skills needed for presentations at conferences or other events. The exposure from a podcast can lead to speaking opportunities. Nevertheless, these points should be rigorously studied in future research to establish quantitative data and associations.

There are limitations to podcasting as well, as mentioned earlier. These are similar to other negative aspects of SoMe, and it can be difficult to grow a podcast audience. Podcasts take time, which many practicing pathologists are greatly limited on outside of their busy clinical, educational, and personal schedules. This can be discouraging for any podcast host. In general, you should not worry too much about the number of downloads at first. Concentrate on creating good, consistent content and developing your skills as a host. The audience will come with time, and promoting content on other SoMe platforms is helpful (e.g., with hyperlinks and short summaries).

## Podcasting national trends

In 2024, there were 546.7 million podcast listeners worldwide, which is a 7.85 % increase from 2023.^23^ By 2027, this number is expected to reach 651.7 million. In the United States (US), the podcast market was expected to reach 160 million listeners in 2024, which is more than double the number of listeners in 2020.^24^ Additionally, Spotify and Apple Podcasts, two of the most popular podcast listening platforms, were expected to grow to 40 and 29 million, respectively. Certain platforms, like Apple Podcasts,^25^ also offer transcripts to make episodes accessible to individuals with hearing impairments. On average, listeners spend about 7 h a week listening to podcasts, and episodes that are 20–40 min long are the most popular.^26^ Notably, podcast listening is not just popular among younger generations: 59 % of 12 to 34-year-olds listen to podcasts monthly, while 27 % of the 55+ demographic group listen at the same frequency.^27^ They are commonly listened to pass the time while multitasking at work, running errands, and completing domestic chores. 73 % of people listen to podcasts on their smartphones, 13 % listen on a desktop or laptop, 28 % listen while driving, and 49 % listen at home.^28^

## Conclusion

Podcasts are audio files released on streaming platforms in episodes, oftentimes part of shows focusing on a broad theme. There are a multitude of pathology podcasts that have been unexplored in medical literature despite their growing popularity. These podcasts are excellent resources for trainees and physicians alike, covering topics from board review to the latest news in the field. Pathology podcasts are inexpensive and accessible tools for learners at all points in their education. Starting a podcast requires very little equipment, just a microphone and editing and publishing platforms, which can reach a wide audience to facilitate learning. There is currently very limited publicly available data regarding trends among podcasters and their audience. Future studies should focus on compiling available data, analyzing their use in MedEd, and elucidating whether there exists a significant benefit for students and trainees who use pathology podcasts as part of their learning.

## Conflict of interest

The authors declare that they have no known competing financial interests or personal relationships that could have appeared to influence the work reported in this article.

## Funding

The article processing fee for this article was funded by an Open Access Award given by the Society of ‘67, which supports the mission of the Association for Academic Pathology to produce the next generation of outstanding investigators and educational scholars in the field of pathology. This award helps to promote the publication of high-quality original scholarship in *Academic Pathology* by authors at an early stage of academic development.
