# Supplementary material for: Pathology podcasts: a growing educational tool
Source: Acad Pathol. 2025 Nov 3;12(4):100227. doi: 10.1016/j.acpath.2025.100227 (PMC12677045; doi:10.1016/j.acpath.2025.100227)
Supplement: Multimedia component 1 [file mmc1.docx]

**Supplemental Table 1.** Additional Countries with PathPod Downloads Over 24 Months (Ending January 2025).

| **Country** | **Total Downloads** | **Percent** |
| --- | --- | --- |
| Australia | 367 | 2.80% |
| Saudi Arabia | 296 | 2.26% |
| Japan | 212 | 1.62% |
| Germany | 206 | 1.57% |
| Syrian Arab Republic | 135 | 1.03% |
| Kuwait | 110 | 0.84% |
| Sweden | 98 | 0.75% |
| France | 90 | 0.69% |
| Brazil | 88 | 0.67% |
| Pakistan | 84 | 0.64% |
| Spain | 72 | 0.55% |
| South Africa | 63 | 0.48% |
| Ireland | 62 | 0.47% |
| Norway | 62 | 0.47% |
| Iran | 59 | 0.45% |
| Hong Kong | 51 | 0.39% |
| China | 47 | 0.36% |
| Oman | 47 | 0.36% |
| Netherlands | 44 | 0.34% |
| Russia | 43 | 0.33% |
| Mexico | 40 | 0.30% |
| Israel | 37 | 0.28% |
| Philippines | 36 | 0.27% |
| Turkey | 36 | 0.27% |
| Puerto Rico | 34 | 0.26% |
| Nepal | 31 | 0.24% |
| Poland | 27 | 0.21% |
| Argentina | 26 | 0.20% |
| Switzerland | 26 | 0.20% |
| Thailand | 26 | 0.20% |
| New Zealand | 25 | 0.19% |
| Cyprus | 21 | 0.16% |
| Iraq | 21 | 0.16% |
| Portugal | 21 | 0.16% |
| Vietnam | 21 | 0.16% |
| Italy | 20 | 0.15% |
| Ethiopia | 18 | 0.14% |
| Romania | 16 | 0.12% |
| Finland | 15 | 0.11% |
| Greece | 15 | 0.11% |
| Czech Republic | 13 | 0.10% |
| Peru | 13 | 0.10% |
| Singapore | 13 | 0.10% |
| Ukraine | 13 | 0.10% |
| Dominican Republic | 12 | 0.09% |
| Colombia | 11 | 0.08% |
| Kenya | 11 | 0.08% |
| South Korea | 11 | 0.08% |
| Myanmar | 10 | 0.08% |
| Taiwan | 10 | 0.08% |
| Jamaica | 9 | 0.07% |
| Paraguay | 9 | 0.07% |
| Sudan | 9 | 0.07% |
| Bangladesh | 7 | 0.05% |
| Belgium | 7 | 0.05% |
| Bolivia | 7 | 0.05% |
| Hungary | 7 | 0.05% |
| Malaysia | 7 | 0.05% |
| Austria | 6 | 0.05% |
| Algeria | 6 | 0.05% |
| Antigua and Barbuda | 5 | 0.04% |
| Chile | 5 | 0.04% |
| Costa Rica | 5 | 0.04% |
| Guatemala | 5 | 0.04% |
| United Arab Emirates | 4 | 0.03% |
| Armenia | 4 | 0.03% |
